# An Investigation on the Therapeutic Potential of Butein, A Tretrahydroxychalcone Against Human Oral Squamous Cell Carcinoma

**DOI:** 10.31557/APJCP.2019.20.11.3437

**Published:** 2019

**Authors:** Devivasha Bordoloi, Javadi Monisha, Monisha Roy, Ganesan Padmavathi, Kishore Banik, Choudhary Harsha, Hong Wang, Alan Prem Kumar, Frank Arfuso, Ajaikumar B Kunnumakkara

**Affiliations:** 1 *Cancer Biology Laboratory, & DAILAB, DBT-AIST International Center for Translational & Environmental Research (DAICENTER), Department of Biosciences and Bioengineering, Indian Institute of Technology Guwahati, Assam, India, *; 2 *Department of Pharmacology, Yong Loo Lin School of Medicine, *; 3 *Singapore Nuclear Research and Safety Initiative, National University of Singapore, Singapore, *; 4 *Stem Cell and Cancer Biology Laboratory, School of Pharmacy and Biomedical Sciences, Curtin Health Innovation Research Institute, Curtin University, Perth, WA 6102, Australia. *

**Keywords:** Oral squamous cell carcinoma, butein, NF-κB, proliferation, survival

## Abstract

**Background:**

Oral squamous cell carcinoma (OSCC) is one of the most predominant cancers in India. With advances in the field of oncology, a number of therapies have emerged; however, they are minimally effective. Consequently, there is a need to develop safe and effective regimens for the treatment of OSCC. Butein, a tetrahydroxychalcone has been found to exhibit potent antioxidant, anti-inflammatory, and also anti-tumor effects against several cancer types. However, its effect on OSCC is not studied yet.

**Methods:**

The effect of butein on the viability, apoptosis, migration and invasion of OSCC cells was evaluated using MTT, colony formation, PI/FACS, live and dead, scratch wound healing, and matrigel invasion assays. Further Western blot analysis was done to evaluate the expression of different proteins involved in the regulation of cancer hallmarks.

**Results:**

This is the first report exemplifying the anti-cancer effect of butein against OSCC. Our results showed that butein exhibited potent anti-proliferative, cytotoxic, anti-migratory, and anti-invasive effects in OSCC cells. It suppressed the expression of NF-κB and NF-κB-regulated gene products such as COX-2, survivin and MMP-9 which are involved in the regulation of different processes like proliferation, survival, invasion, and metastasis of OSCC cells.

**Conclusion:**

Collectively, these results suggest that butein has immense potential in the management of OSCC. Nonetheless, *in vivo* validation is critical before moving to clinical trials.

## Introduction

Oral cancer presents one of the major health concerns of people worldwide, accounting for 2–4% of all cancer cases. It includes a group of neoplasms affecting any region of the oral cavity, pharyngeal regions, and salivary glands. Notably, in some regions, the prevalence of oral cancer is even higher, reaching around 45% in India and 10% in Pakistan of all cancers (Herrero et al., 2003; Pires et al., 2013 ; Markopoulos, 2012). On the basis of epidemiological and clinicopathological standpoints, oral cancer can be divided into three distinct classes, such as carcinomas of the oral cavity proper, carcinomas of the lip vermilion, and carcinomas arising in the oropharynx. It is more common among older and middle-aged people compared to younger adults. Further, recent reports suggest that intraoral and oropharyngeal tumors occur more commonly among men than women, with a male: female ratio of 2:1. Consumption of tobacco, alcohol, chronic use of betel quid, human papillomavirus, and oral lichen planus have been identified as major risk factors of oral cancer (Neville and Day, 2002; Ram et al., 2011). It has been estimated that more than 90% of all oral cancers are oral squamous cell carcinomas (OSCC) (Pires et al., 2013). Notably, despite the tremendous advancements made in surgery, radiotherapy, and chemotherapy, the survival rate of oral cancer has not shown any appreciable improvement; rather, it remains quite low at around 50-55%. This is primarily due to current therapeutic regimens being of minimal efficacy and being associated with diverse adverse reactions. Further, they are very expensive and hence beyond the reach of the vast majority of the world’s population. This necessitates discovering alternate regimens that are effective, devoid of extreme toxicities, and inexpensive for treating this neoplasm (Bordoloi et al., 2016; Kunnumakkara et al., 2017; Kunnumakkara et al., 2019a). 

**Figure 1:**
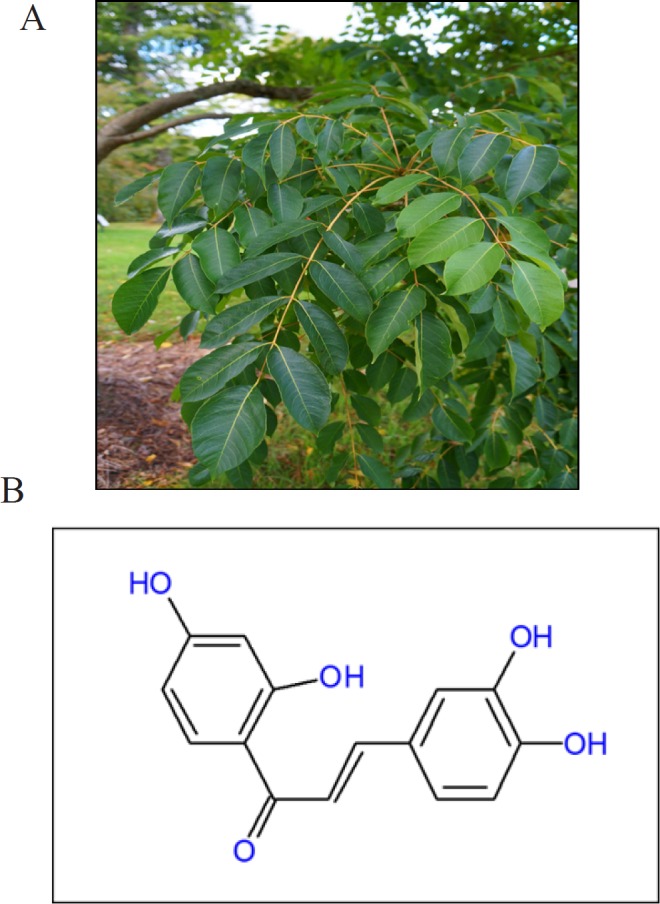
A, *Toxicodendron vernicifluum*, the plant from which Butein was first isolated; B, Chemical structure of Butein

Numerous epidemiological, clinical, and experimental studies suggest that natural products possess enormous therapeutic potential for the prevention and treatment of different malignancies (Deorukhkar et al., 2007; Pandey et al., 2007; Shanmugam et al., 2011a; Yang et al., 2013; Dai et al., 2015a; Hsieh et al., 2015; Roy et al., 2016; Shanmugam et al., 2016; Yarla et al., 2016; Harsha et al., 2017; Hasanpourghadi et al., 2017; Shanmugam et al., 2017; Banik et al., 2018; Jayasooriya et al., 2018; Khwairakpam et al., 2018; Kunnumakkara et al., 2019b). For instance, various plant polyphenols have been suggested to inhibit tumorigenesis and carcinogenesis of skin, liver, colon and lung cells (Lambert et al., 2005; Li F et al., 2010; Tan et al., 2010; Rajendran et al., 2011a; Rajendran et al., 2011b; Chan et al., 2012; Rajendran et al., 2012; Manu et al., 2013; Subramaniam et al., 2013; Sethi et al., 2014; Siveen et al., 2014a; Tang et al., 2014; Dai et al., 2015b; Lee et al., 2015; Amararathna et al., 2016; Dai et al., 2016; Ko et al., 2018). Butein is a one such potent polyphenol that was first isolated from *Toxicodendron vernicifluum* (also known as *Rhus verniciflua*), (Figure 1) with enormous medicinal values that have been utilized since ancient times (Chua et al., 2010; Padmavathi et al., 2015; Song et al., 2016). This plant has been used in the treatment of gastritis, atherosclerosis, and various other diseases such as inflammatory diseases, hepatic disorders, cancers, bleeding, and cough, and has also been used as a pain killer, antioxidant, and antibacterial agent (Padmavathi et al., 2015). It is present in numerous other plants as well, which include the stem-bark of cashews (*Semecarpus anacardium*), and herbs such as *Caragana jubata* and the heartwood of *Dalbergia odorifera* (Moon et al., 2010b). Research over the past few decades has revealed butein to be a potent, multi-targeted flavonoid. It exhibits anti-inflammatory, anti-platelet, anti-restenosis, anti-diabetic, and anti-nephritic activities, exemplifying its multi-targeting potential (Padmavathi et al., 2015). Further, it exhibits anti-tumor activity against a variety of human tumor cells, including colon carcinoma, osteosarcoma, breast carcinoma, hepatocarcinoma, and lymphoma (Jayasooriya et al., 2018). Butein was shown to alter the expression and activity of several genes, transcription factors, enzymes, and proteins involved in important cellular processes essential for tumor initiation, progression, and chemoresistance (Padmavathi et al., 2017). The major molecular target affected by butein treatment in most of the diseases investigated is the transcription factor nuclear factor κB (NF-κB) (Padmavathi et al., 2017). Over the last decade, NF-κB became a major target in drug discovery due to its key role in cancer development, cell proliferation and survival, inflammation, and immune responses (Ahn et al., 2007; Sethi et al., 2008; Sethi et al., 2009; Li and Sethi, 2010; Orlikova et al., 2012; Sethi et al., 2012; Shin et al., 2014; Li et al., 2015b; Monisha et al.,2016; Monisha et al., 2017; Ningegowda et al., 2017; Pires et al., 2018; Mohan et al., 2018; Puar et al., 2018). Hence, in this study we aimed to evaluate the effect of butein on NF-κB and its regulated gene products in OSCC cells.

## Materials and Methods

*Reagents*

Butein was obtained from TCI, Japan. A 50 mM solution of butein was prepared in dimethyl sulfoxide (DMSO), stored as small aliquots at -20°C, and then diluted as needed in cell culture medium. Penstrep, DMEM medium, and fetal bovine serum were obtained from Gibco, USA. MTT and Propidium iodide were obtained from Sigma Aldrich and DMSO used was from Merck Life Science Pvt. Ltd. Antibodies against p65, MMP-9, COX-2, and survivin were obtained from Cell signaling technologies, USA. The live and dead assay kit was obtained from Life technologies. The Matrigel invasion assay kit was obtained from Corning, New York, USA.

*Cell lines*

Cell lines SAS and KB (human oral squamous cancer cells) were obtained from NCCS (Pune, India). The cells were cultured in DMEM medium supplemented with 10% fetal bovine serum and 1 % Penstrep. 

*DPPH free radical scavenging assay*

2,2-Diphenyl–1–picrylhydrazyl (DPPH∙) was used to measure the free radical scavenging activity of butein. DPPH stable free radicals are reduced to DPPH-H, leading to discoloration from purple to yellow and consequently a decrease in absorbance. The degree of discoloration indicates the scavenging potential of the antioxidant compounds (Wang and Wink, 2016). To perform this assay, 0.1 mM solution of DPPH in methanol was prepared and 100μl of this solution was added to different concentrations of butein (0, 25, 50 and 100 µM). After 30 minutes of incubation, the absorbance was measured at 517nm using a microplate reader (TECAN Infinite 200 PRO multimode reader). Curcumin at various concentrations (0, 25, 50, and 100 μM) was used as a reference compound. The lower the absorbance of the reaction mixture indicates a higher free radical scavenging activity. The capability to scavenge DPPH free radicals was calculated using the following equation: Radical Scavenging (%)=((OD of Control- OD of Sample)/OD of Control)×100, where OD is optical density (Banothu et al., 2018). The graph was plotted as the percentage of DPPH inhibition vs. concentration.

*Viability assay*

The effect of butein on the viability of OSCC cells was determined using an MTT assay. Briefly, 2,000 cells were seeded in 96 well plates and incubated for 24h at 37°C and then treated with different concentrations of butein (0, 1, 10, 25, 50, and 100 µM). The MTT assay was performed at 24, 48, and 72h. 10µl of 5mg/ml of MTT solution was added to each well and incubated for 2h. After that the culture medium was removed and 100µl of DMSO was added to all the wells and incubated at room temperature for 1h to dissolve the MTT-formazan product. Finally, absorbance of the colored solution was measured with a microplate reader (TECAN Infinite 200 PRO multimode reader) at 570 nm. The inhibition caused by butein on the growth of SAS and KB cells was determined by the percentage of viability. 

*Colony formation assay*

The clonogenic potential of butein treated OSCC cells was determined using a colony formation assay. Briefly, cells were seeded in 6-well plates at low density (~1,000 cells per well) and then treated with the indicated concentrations of butein for 24h. The medium was replaced with fresh medium and the cells were cultured for 10 days. The plates were then washed with PBS and stained with crystal violet (SRL Pvt. Ltd., India). The images of each well were scanned, the individual clone types were identified, and survival fraction was determined. 

*Propidium iodide flow cytometric assay*

Propidium iodide (PI), a fluorescent dye that intercalates with nucleic acids to give red fluorescence, was used to measure cell viability in flow cytometry. SAS and KB cells were seeded in 6-well plates at a concentration of 5x10^4^ cells per well, incubated for 24h, and then treated with the indicated concentrations of butein for 24, 48, and 72h. After that, all the cells were collected, washed with PBS, and 5 µl of 1mg/ml PI was added. After incubation for 10 min, the effect of butein on the cells was analyzed using FACS (FACS Calibur™, BD Biosciences). Live cells have an intact cell membrane and hence are PI impermeable and emit less fluorescence, whereas dead cells emit a high red fluorescence due to the presence of a damaged plasma membrane.

*Live/Dead assay*

In order to measure apoptosis, we performed a Live/Dead assay, which determines intracellular esterase activity and plasma membrane integrity. Calcein-AM, a non-fluorescent polyanionic dye, is retained by live cells in which it produces intense green fluorescence through enzymatic (esterase) conversion. In addition, an ethidium homodimer enters the cells with damaged membranes and binds to nucleic acids, thereby producing a bright red fluorescence in dead cells (Lima et al., 2015). Briefly, 2x10^4^ cells were incubated with the indicated concentrations of butein for 72h at 37°C. Cells were then stained with the Live/Dead reagent (5 µM ethidium homodimer and 5 µM calcein-AM) and incubated at 37°C for 20 min. Cells were analyzed under an inverted fluorescence microscope (Nikon, Eclipse TS100, Japan).

*Scratch wound healing assay*

This assay is performed to evaluate cell migration, where an artificial gap is created in a confluent cell monolayer and then migration of the cells from the edges over time is observed. The extent of scratch wound healing and the scratch wound healing rate denote migration characteristics of the cells. 6x10^5^ OSCC cells were seeded in 6-well cell culture plates and cultured to a confluent monolayer. After the monolayer was formed, the medium was replaced with serum free medium and the cells were incubated for 8h. Then the medium was removed and a pipette tip (200 μl) was used to scratch a wound in the midline of the culture well, and then the cells were washed twice with PBS to remove any debris. Cells were then treated with the indicated concentration of butein. The migration of the cells was evaluated by observing the difference in the area of the scratch wounds using an inverted microscope (Nikon T1-SM, Japan). Images were taken at 0 and 24h and analyzed using Image J software. As KB cells mostly formed colonies, no monolayer was obtained and hence this assay was performed for SAS cells only.

*Invasion assay*

Invasion through the extracellular matrix is a crucial step in tumor metastasis. The invasive potential of butein-treated OSCC cells was carried out using a Boyden chamber assay. OSCC cells were serum starved for 18h before seeding to transwell migration chambers. 24-well, 8mm pore transwell inserts (Corning, USA) pre-coated with Matrigel were used. Post serum starvation, the cells were trypsinized and were seeded at a concentration of 5x10^4^ cells in the upper chamber of the transwell insert in 500µl of serum free medium and then treated with the indicated concentrations of butein. Then 750µl of medium containing 10% FBS was added to the lower chamber as a chemo-attractant. Cells were then incubated for another 24h at 37°C. The non-migrating cells on the upper surface of the membrane were then scraped off with cotton swabs. The migrated cells at the bottom of the transwell insert were fixed in 70% ethanol and were stained with crystal violet solution. Stained cells were visualized under an inverted microscope (Nicon Eclipse TS100), the membrane was dissolved in 1% SDS solution at 37°C for 1h and absorbance was read at 595nm using a microplate reader (TECAN Infinite 200 PRO multimode reader).

*Western blot analysis*

To determine the effect of butein on p65 phosphorylation, cytoplasmic and nuclear extracts were prepared. For the detection of anti-apoptotic, invasion, and metastasis markers, whole cell extracts were prepared by subjecting butein-treated cells to lysis in lysis buffer (20mM HEPES, 2mM EDTA, 250mM NaCl, 0.1% NP40) in the presence of protease inhibitors (2µg/ml Leupeptin hemisulfate, 2µg/ml aprotinin, 1mM PMSF, 1mM DTT). Lysates were spun at 13,000 g for 10 min to remove insoluble material. Supernatants were collected and kept at -80°C. Both cytosolic or nuclear extracts and whole cell lysates were resolved by SDS-PAGE. After electrophoresis, the proteins were electro transferred to nitrocellulose membranes, blotted with the relevant antibodies, and detected using an Opti blot ECL Detection Kit (Abcam, USA).

*Statistical analysis*

All data were derived from at least three independent experiments. The blots were visualized with the chemi-doc system (BIORAD). Images were captured and analyzed using Image-Lab software. Image J software was also used for the analysis of the images. Statistical analyses were conducted using Student’s t-test, and values are presented as mean ± standard deviation (SD). A value of P < 0.05 was considered statistically significant.

## Results

The study was designed to evaluate the anti-cancer effect of butein on human OSCC cells. Additionally, we attempted to explore potential mechanism(s) through which butein exerts its effect on OSCC cells. 

*Butein exhibited radical scavenging activity*

**Figure 2:**
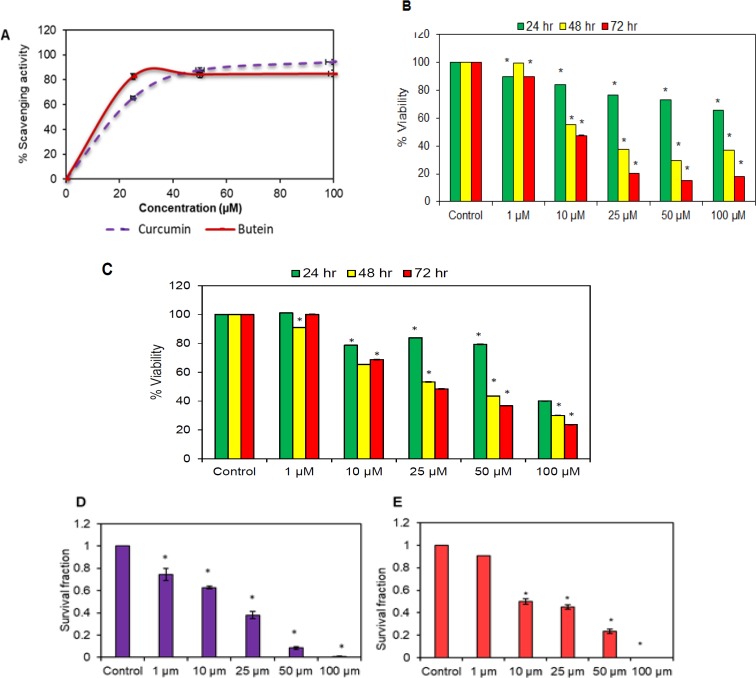
A, Anti-oxidant effect of butein evaluated using DPPH assay. The graph shows % scavenging activity of the indicated concentrations of butein and curcumin. Results are represented as Mean ± SD of three independent experiments. Cell viability of SAS cells (B) and KB cells (C) was determined with the help of MTT assay. After 24, 48 and 72 hr drug exposure with the indicated concentrations of butein, cells were incubated with MTT for 2 hr. Absorbance values were measured at 570 nm. Colony formation assay of SAS cells (D) and KB cells (E) was done to investigate the suppressive effect of butein on the clonogenic potential of human OSCC cells. Data are presented as mean ± SD of three independent experiments, *p < 0.05 vs. control

A DPPH assay was conducted to assess the radical scavenging effect of butein. Curcumin (Akay Flavours and Aromatics Pvt. Ltd., India) was used as a positive control. The ability to scavenge the DPPH radicals was calculated and the results indicate that butein exhibits a strong radical scavenging effect (Figure 2A).

*Butein reduced the viability of OSCC cells in vitro*

We examined whether butein could affect the viability of human OSCC cells by using an MTT assay and colony formation assay. Butein was found to effectively reduce the viability of SAS (Figure 2B) and KB (Figure 2C) cells. Overall, this activity of butein was dose-dependent as well as time-dependent. Further, a colony formation assay was performed to investigate the long-term effect of butein on the proliferation of OSCC cells. The colony-forming ability of both SAS (Figure 2D) and KB (Figure 2E) cells was reduced after exposure to butein. Additionally, the reduction was even more remarkable at higher concentrations i.e. 50 and 100 µM.

*Butein induced apoptosis in OSCC cells*

**Figure 3:**
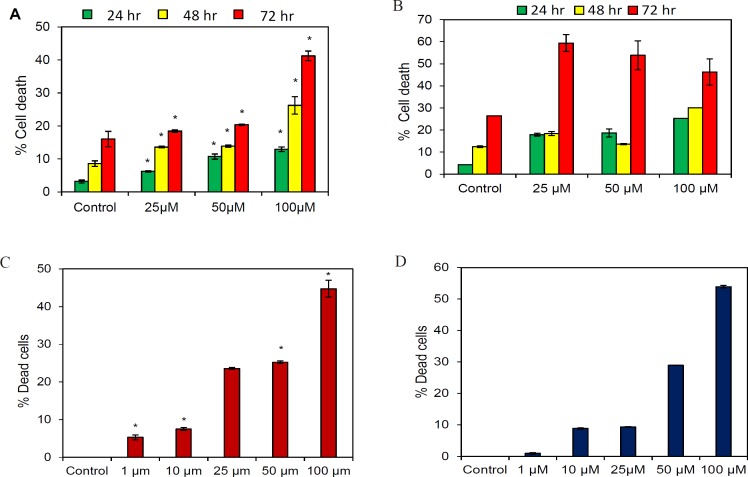
Butein Induces Cytotoxic Effect to the Oral Cancer Cells. (A): SAS and (B): KB cells were treated with the indicated concentrations of butein for 24, 48 and 72 h, followed by PI staining and FACS analysis for the cell death profile. Live and dead assay was performed to evaluate the apoptotic effect of the indicated concentrations of butein on OSCC cells. Quantification of the number of live (green) and dead cells (red) was done with the help of Image J software in butein treated (C) SAS cells and (D) KB cells. Results presented are mean ± SD of three independent experiments, *p < 0.05 vs. control

The apoptotic effect of butein was evaluated using PI FACS and live and dead assay. Both SAS and KB cells were treated with different concentrations of butein for 24, 48, and 72 hr following which apoptosis was determined by flow cytometry analysis. As shown in Figure 3, treatment of SAS (Figure 3A) and KB (Figure 3B) cells with different concentrations of butein for different time periods resulted in cell death, which was found to be dose- as well as time-dependent.

Further, in a live and dead assay, butein treatment showed a dose-dependent toxic effect on OSCC cells. The effect was more pronounced at concentrations beyond 25 µM. This dose-dependent reduction of viable cells is in agreement with the results of the PI FACS analysis (Figures 3C and D).

*Butein inhibited the migration and invasion of OSCC cells*

**Figure 4.**
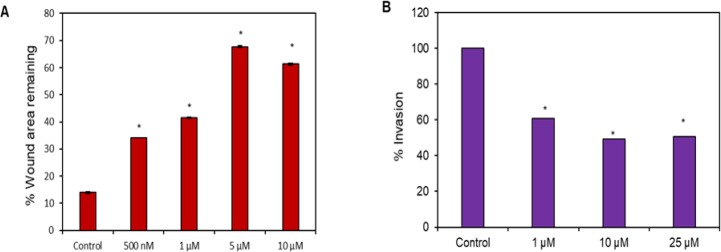
(A), Effect of butein on the migration of SAS cells *in vitro*. SAS cells were scratch-wounded and then treated with the indicated concentrations of butein followed by recording of wound areas at 0 and 24 hr. % wound area remaining was calculated using Image J software; (B), Anti-invasive effect of butein on SAS cells using a matrigel invasion assay where transwell inserts pre-coated with matrigel were used to measure the *in vitro* invasiveness. The % invaded cells were significantly reduced by butein treatment. Results are presented as mean ± SD of three independent experiments, *p < 0.05 vs. control

Cell migration was assessed by quantifying the percentage of wound closure in the scratch wound-healing assay. Butein treated cells exhibited lower values of migration ability with respect to those obtained by the untreated cells. In addition, butein was found to inhibit the migration of SAS cells dose-dependently (Figure 4A). We also examined the effect of butein on the invasive properties of SAS cells using a Matrigel invasion assay (Figure 4B). The results demonstrated that the invasion of cells was very low after treatment with butein compared to the untreated cells. Notably, the anti-invasive effect of butein was found to increase proportionately with the increased concentrations of butein. At 25 µM concentration of butein treatment, 50 % inhibition in the invasive capacity of SAS cells was observed.

*Butein inhibited NF-κB activation and NF-κB-regulated proteins associated with proliferation, survival, invasion, and metastasis of OSCC cells*

**Figure 5:**
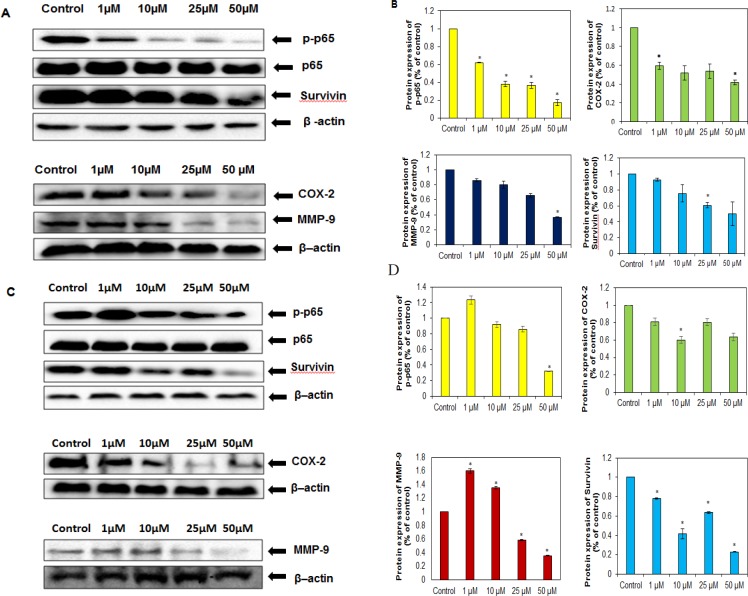
Effect of Butein on NF-κB and NF-κB Regulated Gene Products. Representative western blots showing p65, phospho p65, survivin, COX-2 and MMP-9 in (A) SAS and (C) KB cell lysates after treating with the indicated concentrations of butein. Densitometric analysis of phospho p65, survivin, COX-2 and MMP-9 in (B) SAS cells and (D) KB cells. Data are expressed as mean ± SD (n=3), * denotes P<0.05 vs. Control

As NF-κB has been linked with both proliferation and chemoresistance in cancer cells (Li and Sethi, 2010; Kannaiyan et al., 2011; Manu et al., 2014; Siveen et al., 2014b; Li et al., 2015b; Manu et al., 2018), we further examined whether butein could inhibit constitutive activation of NF-κB in OSCC cells. Our results showed that this flavonoid inhibited the activation of NF-κB. Further, we examined whether butein downregulated the expression of the proteins involved in proliferation, survival, invasion, and metastasis in both SAS (Figure 5A) and KB (Figure 5C) cells. The results showed that this compound downregulated the expression of survivin in both cell lines (Figures 5A-D). In addition, the expression of cyclooxygenase-2; COX-2 and matrix metalloproteinase 9; MMP-9 were also suppressed by butein in SAS cells (Figures 5A and 5B). Further, in KB cells, butein downregulated the expression of COX-2; whereas in the case of MMP-9, downregulation was observed upon treating with 25 and 50 µM of butein (Figures 5C and 5D).

## Discussion

Butein is a biologically active flavonoid, which is known to have therapeutic potential against various cancers (Padmavathi et al., 2015; Jayasooriya et al., 2018). It targets different proteins such as EGFR, COX-2, STAT3, ERK, JNK, Akt, p38, caspases, Bax etc. which regulate different processes in cancer cells like survival, proliferation, invasion, metastasis, chemoresistance, radiation resistance, and cell death (Padmavathi et al., 2015). Notably, this is the first report to describe the anti-cancer potential of butein against OSCC. Our results showed this flavonoid to effectively modulate the viability, clonogenic potential, apoptosis, migration as well as invasion of OSCC cells plausibly through the involvement of NF-κB and NF-κB regulated gene products which play critical role in regulating diverse cancer hallmarks. In this study, it was observed that butein showed high antioxidant activity. In line with this result obtained, previous report has also indicated the powerful antioxidant role of butein against lipid and LDL peroxidation via free radical scavenging processes and metal ion chelation (Cheng et al., 1998). Antioxidants are known to mediate the key activities of cellular integrity maintenance and thus maintains the homeostasis of the host immune system. The genomic integrity of a cell is determined by the balance of pro-oxidants and the levels of antioxidants and an aberration in this balance can alter the normal cellular signaling cascades (Thyagarajan et al., 2018). Further, the alterations in the cellular signaling pathway can initiate the process of neocarcinogenesis (Monisha et al., 2017; Roy et al., 2017). In addition, as mentioned above, the current investigation suggested that the treatment of butein caused antiproliferative and cytotoxic effects against OSCC cells. Similarly, the preceding reports have also suggested the same activities against different cancer cells such as breast, blood, liver, cervical etc. (Yang et al., 2012; Liao et al., 2018; Tang et al., 2016; Yang et al., 2018). For instance, Yang et al., (2018) reported that butein reduced the viability of cervical cancer cells through a pro-apoptotic effect by blocking inhibitor of apoptosis (IAP) proteins and activating extrinsic as well as intrinsic pro-apoptotic cascades. Further, it inhibited the proliferation and survival of HCC ells effectively (Liao et al., 2018; Zhou et al., 2018). In addition, butein was also reported to exert its antiproliferative and pro-apoptotic effect through inhibition of NF-κB, AP-1 and Akt signaling in HTLV-1-infected T cells in both *in vitro* and *in vivo* settings clearly indicating its therapeutic potential against adult T-cell leukemia/lymphoma (Ishikawa et al., 2017). Besides, this flavonoid induced apoptosis and arrest at the G2/M phase of cell cycle through PERK/eIF-2α/CHOP pathway dependent ROS generation (Di et al., 2019). 

This study also showed that butein inhibited the migration and invasion of OSCC cells. In line with our findings, Lai et al., (2015) also reported that butein inhibited the migration of B16F10 melanoma cells in a concentration-dependent manner. Further it exerted a dose-dependent effect on focal adhesion kinase, Akt, and ERK phosphorylation in B16F10 cells. In addition, its treatment led to the inhibition of cell adhesion, migration and invasion of NSCLC cells (Di et al., 2019). Zhang et al., (2008) also reported this compound to inhibit the migration as well as invasion of bladder cancer cells by modulating ERK1/2 and NF-kB signaling. Further, in case of HCC as well, butein was found to inhibit the migration and invasion of SK-HEP-1 human HCC cells via ERK, JNK, p38, and uPA signaling cascades (Ma et al., 2011). Another study reported this compound to be a novel inhibitor of CXCR4 and thus found to hold immense potential in suppressing metastasis in pancreatic cancer and also in case of breast cancer, the most predominant cancer type among women across the world (Chua et al., 2010; Thakur et al., 2018). Further, a detailed analysis of the underlying mechanism of action of butein was also carried out in this study. Notably, the recent studies reported NF-κB and NF-κB regulated proteins to be the major targets of this compound (Padmavathi et al., 2015; Padmavathi et al., 2017). It was found to exhibit the effect by suppressing NF-κB in different cancer cell lines such as breast, bladder, hepatoma, lung, leukemia, pancreatic, and prostate (Pandey et al., 2007; Zhang et al., 2008; Chua et al., 2010; Moon et al., 2010a, Moon et al., 2010b; Ishikawa et al., 2017). It has been well established that NF-κB plays a pivotal role in inflammatory and immune responses and regulates the expression of a variety of important genes associated with different hallmarks of cancer (Manu et al., 2011; Shanmugam et al., 2011b; Manu et al., 2012; Li et al., 2013; Li et al., 2015a; Li et al., 2015b; Monisha et al., 2017; Ningegowda et al., 2017) . However, the effect of butein on NF-κB, as well as diverse gene products regulated by NF-κB, in human OSCC cells has not been previously elucidated. Our results showed that butein suppressed the expression of *COX-2*, survivin and MMP-9. COX-2 is known to be involved in the modulation of cell proliferation and apoptosis in different tumors (Zarghi et al., 2011). Further, survivin which belongs to IAP protein family is involved in inhibiting caspases as well as blocking cell death (Jaiswal et al., 2015). MMP-9 is strongly involved in the regulation of cancer cell invasion, metastasis and angiogenesis (Huang, 2018). Further, butein also suppressed the activation of NF-κB in OSCC cells. These results are in agreement with the previous reports of butein on different tumor cells. For instance, a study conducted by Pandey et al., (2007) demonstrated that the anti-tumor effect exerted by butein was mediated via inhibition of IKK, resulting in the suppression of the NF-κB pathway. Another study showed that this chalcone caused repression in the invasion of bladder cancer cells through NF-κB and ERK1/2 signaling cascades (Zhang et al., 2008). Further, butein inhibited tumor necrosis factor-related apoptosis-inducing ligand (TRAIL)-mediated activation of NF-kB in human hepatoma cells (Moon et al., 2010b). In addition, butein inhibited the invasion and angiogenesis in prostate cancer cells through blockade of NF-κB activity (Moon et al., 2010a). In the case of breast and pancreatic cancer cells, butein downregulated chemokine receptor CXCR4 expression and acted via suppression of NF-κB (Chua et al., 2010). 

Taken together, this plant polyphenol, ‘butein’ has been found to exhibit profound anti-proliferative, cytotoxic, anti-migratory, and anti-invasive effects via modulation of various gene products such as COX-2, survivin, and MMP-9. As mentioned earlier, these genes play inevitably important roles in the proliferation, survival, invasion, and metastasis of cancer cells, including OSCC. This is the first report that shows the anti-cancer effect of butein along with the possible mechanisms involved. As these gene products are regulated by NF-κB, it is a clear indication that butein exerts its anti-cancer effect via modulation of the NF-κB signaling pathway. However, further *in vivo* studies and clinical validation are warranted to bring this safe and efficacious compound as a forefront therapeutic option for the successful management of OSCC. 

## Data Availability

The data used to support the findings of this study are included within the article.

## References

[B1] Ahn KS, Sethi G, Aggarwal BB 2007) Nuclear factor-kappa B: from clone to clinic. Curr Mol Med.

[B2] Amararathna M, Johnston MR, Rupasinghe HP (2016). Plant polyphenols as chemopreventive agents for lung cancer. Int J Mol Sci.

[B3] Banik K, Harsha C, Bordoloi D (2018). Therapeutic potential of gambogic acid, a caged xanthone, to target cancer. Cancer Lett.

[B4] Banothu V, Neelagiri C, Adepally U, Lingam J, Bommareddy K (2017). Phytochemical screening and evaluation of in vitro antioxidant and antimicrobial activities of the indigenous medicinal plant Albizia odoratissima. Pharm Biol.

[B5] Bordoloi D, Roy NK, Monisha J, Padmavathi G, Kunnumakkara AB (2016). Multi-targeted agents in cancer cell chemosensitization: What we Learnt from curcumin Thus Far. Recent Pat Anticancer Drug Discov.

[B6] Chan AT, Arber N, Burn J (2012). Aspirin in the chemoprevention of colorectal neoplasia: an overview. Cancer Prev Res (Phila).

[B7] Cheng ZJ, Kuo SC, Chan SC, Ko FN, Teng CM (1998). Antioxidant properties of butein isolated from Dalbergia odorifera. Biochim Biophys Acta.

[B8] Chua AW, Hay HS, Rajendran P (2010). Butein downregulates chemokine receptor CXCR4 expression and function through suppression of NF-kappaB activation in breast and pancreatic tumor cells. Biochem Pharmacol.

[B9] Dai X, Ahn KS, Kim C (2015b). Ascochlorin, an isoprenoid antibiotic inhibits growth and invasion of hepatocellular carcinoma by targeting STAT3 signaling cascade through the induction of PIAS3. Mol Oncol.

[B10] Dai X, Ahn KS, Wang LZ (2016). Ascochlorin enhances the sensitivity of doxorubicin leading to the reversal of epithelial-to-mesenchymal transition in hepatocellular carcinoma. Mol Cancer Ther.

[B11] Dai X, Zhang J, Arfuso F (2015a). Targeting TNF-related apoptosis-inducing ligand (TRAIL) receptor by natural products as a potential therapeutic approach for cancer therapy. Exp Biol Med (Maywood).

[B12] Deorukhkar A, Krishnan S, Sethi G, Aggarwal BB (2007). Back to basics: how natural products can provide the basis for new therapeutics. Expert Opin Investig Drugs.

[B13] Di S, Fan C, Ma Z (2019). PERK/eIF-2α/CHOP pathway dependent ROS generation mediates butein-induced non-small-cell lung cancer apoptosis and G2/M phase arrest. Int J Biol Sci.

[B14] Harsha C, Banik K, Bordoloi D, Kunnumakkara AB (2017). Antiulcer properties of fruits and vegetables: A mechanism based perspective. Food Chem Toxicol.

[B15] Hasanpourghadi M, Looi CY, Pandurangan AK, Sethi G, Wong WF (2017). Phytometabolites targeting the warburg effect in cancer cells: A mechanistic review. Curr Drug Targets.

[B16] Herrero R, Castellsague X, Pawlita M (2003). Human papillomavirus and oral cancer: the International Agency for Research on Cancer multicenter study. J Natl Cancer Inst.

[B17] Hsieh YS, Yang SF, Sethi G, Hu DN (2015). Natural bioactives in cancer treatment and prevention. Biomed Res Int.

[B18] Huang H (2018). Matrix Metalloproteinase-9 (MMP-9) as a cancer biomarker and MMP-9 biosensors: Recent advances. sensors (Basel).

[B19] Ishikawa C, Senba M, Mori N (2017). Butein inhibits NF-kappaB, AP-1 and Akt activation in adult T-cell leukemia/lymphoma. Int J Oncol.

[B20] Jaiswal PK, Goel A, Mittal RD (2015). Survivin: A molecular biomarker in cancer. Indian J Med Res.

[B21] Jayasooriya R, Molagoda IMN, Park C (2018). Molecular chemotherapeutic potential of butein: A concise review. Food Chem Toxicol.

[B22] Kannaiyan R, Hay HS, Rajendran P (2011). Celastrol inhibits proliferation and induces chemosensitization through down-regulation of NF-kappaB and STAT3 regulated gene products in multiple myeloma cells. Br J Pharmacol.

[B23] Khwairakpam AD, Bordoloi D, Thakur KK (2018). Possible use of Punica granatum (Pomegranate) in cancer therapy. Pharmacol Res.

[B24] Ko JH, Nam D, Um JY, Jung SH, Sethi G (2018). Bergamottin suppresses metastasis of lung cancer cells through abrogation of diverse oncogenic signaling cascades and epithelial-to-mesenchymal transition. Molecules.

[B25] Kunnumakkara AB, Bordoloi D, Harsha C (2017). Curcumin mediates anticancer effects by modulating multiple cell signaling pathways. Clin Sci (Lond).

[B26] Kunnumakkara AB, Bordoloi D, Sailo BL (2019a). Cancer drug development: The missing links. Exp Biol Med (Maywood).

[B27] Kunnumakkara AB, Harsha C, Banik K (2019b). Is curcumin bioavailability a problem in humans: lessons from clinical trials. Expert Opin Drug Metab Toxicol.

[B28] Lai YW, Wang SW, Chang CH (2015). Butein inhibits metastatic behavior in mouse melanoma cells through VEGF expression and translation-dependent signaling pathway regulation. BMC Complement Altern Med.

[B29] Lambert JD, Hong J, Yang GY, Liao J, Yang CS (2005). Inhibition of carcinogenesis by polyphenols: evidence from laboratory investigations. Am J Clin Nutr.

[B30] Lee JH, Kim C, Sethi G, Ahn KS (2015). Brassinin inhibits STAT3 signaling pathway through modulation of PIAS-3 and SOCS-3 expression and sensitizes human lung cancer xenograft in nude mice to paclitaxel. Oncotarget.

[B31] Li F, Fernandez PP, Rajendran P, Hui KM, Sethi G (2010). Diosgenin, a steroidal saponin, inhibits STAT3 signaling pathway leading to suppression of proliferation and chemosensitization of human hepatocellular carcinoma cells. Cancer Lett.

[B32] Li F, Sethi G (2010). Targeting transcription factor NF-kappaB to overcome chemoresistance and radioresistance in cancer therapy. Biochim Biophys Acta.

[B33] Li F, Shanmugam MK, Chen L (2013). Garcinol, a polyisoprenylated benzophenone modulates multiple proinflammatory signaling cascades leading to the suppression of growth and survival of head and neck carcinoma. Cancer Prev Res (Phila).

[B34] Li F, Shanmugam MK, Siveen KS (2015a). Garcinol sensitizes human head and neck carcinoma to cisplatin in a xenograft mouse model despite downregulation of proliferative biomarkers. Oncotarget.

[B35] Li F, Zhang J, Arfuso F (2015b). NF-kappaB in cancer therapy. Arch Toxicol.

[B36] Liao W, Liu J, Zhang D, Huang W, Chen R (2018). Butein inhibited in vitro hexokinase-2-mediated tumor glycolysis in hepatocellular carcinoma by blocking epidermal growth factor receptor (EGFR). Med Sci Monit.

[B37] Lima RA, Candido EB, de Melo FP (2015). Gene expression profile of ABC transporters and cytotoxic effect of ibuprofen and acetaminophen in an epithelial ovarian cancer cell line in vitro. Rev Bras Ginecol Obstet.

[B38] Ma CY, Ji WT, Chueh FS (2011). Butein inhibits the migration and invasion of SK-HEP-1 human hepatocarcinoma cells through suppressing the ERK, JNK, p38, and uPA signaling multiple pathways. J Agric Food Chem.

[B39] Manu KA, Shanmugam MK, Li F (2014). Simvastatin sensitizes human gastric cancer xenograft in nude mice to capecitabine by suppressing nuclear factor-kappa B-regulated gene products. J Mol Med (Berl).

[B40] Manu KA, Shanmugam MK, Ong TH (2013). Emodin suppresses migration and invasion through the modulation of CXCR4 expression in an orthotopic model of human hepatocellular carcinoma. PLoS One.

[B41] Manu KA, Shanmugam MK, Rajendran P (2011). Plumbagin inhibits invasion and migration of breast and gastric cancer cells by downregulating the expression of chemokine receptor CXCR4. Mol Cancer.

[B42] Manu KA, Shanmugam MK, Ramachandran L (2012). First evidence that gamma-tocotrienol inhibits the growth of human gastric cancer and chemosensitizes it to capecitabine in a xenograft mouse model through the modulation of NF-kappaB pathway. Clin Cancer Res.

[B43] Manu KA, Shanmugam MK, Ramachandran L (2018). Corrigendum on “Isorhamnetin augments the anti-tumor effect of capeciatbine through the negative regulation of NF-kappaB signaling cascade in gastric cancer”. Cancer Lett.

[B44] Mohan CD, Anilkumar NC, Rangappa S (2018). Novel 1,3,4-oxadiazole induces anticancer activity by targeting NF-kappaB in hepatocellular carcinoma cells. Front Oncol.

[B45] Monisha J, Padmavathi G, Roy NK (2016). NF-kappaB blockers gifted by mother nature: Prospectives in cancer cell chemosensitization. Curr Pharm Des.

[B46] Monisha J, Roy NK, Bordoloi D (2017). Nuclear factor Kappa B: A potential target to persecute head and neck cancer. Curr Drug Targets.

[B47] Moon DO, Choi YH, Moon SK, Kim WJ, Kim GY (2010a). Butein suppresses the expression of nuclear factor-kappa B-mediated matrix metalloproteinase-9 and vascular endothelial growth factor in prostate cancer cells. Toxicol In Vitro.

[B48] Moon DO, Kim MO, Choi YH, Kim GY (2010b). Butein sensitizes human hepatoma cells to TRAIL-induced apoptosis via extracellular signal-regulated kinase/Sp1-dependent DR5 upregulation and NF-kappaB inactivation. Mol Cancer Ther.

[B49] Neville BW, Day TA (2002). Oral cancer and precancerous lesions. CA Cancer J Clin.

[B50] Ningegowda R, Shivananju NS, Rajendran P (2017). A novel 4,6-disubstituted-1,2,4-triazolo-1,3,4-thiadiazole derivative inhibits tumor cell invasion and potentiates the apoptotic effect of TNFalpha by abrogating NF-kappaB activation cascade. Apoptosis.

[B51] Orlikova B, Schnekenburger M, Zloh M (2012). Tasdemir D. Natural chalcones as dual inhibitors of HDACs and NF-kappaB. Oncol Rep.

[B52] Padmavathi G, Rathnakaram SR, Monisha J (2015). Potential of butein, a tetrahydroxychalcone to obliterate cancer. Phytomedicine.

[B53] Padmavathi G, Roy NK, Bordoloi D (2017). Butein in health and disease: A comprehensive review. Phytomedicine.

[B54] Pandey MK, Sandur SK, Sung B (2007). Butein, a tetrahydroxychalcone, inhibits nuclear factor (NF)-kappaB and NF-kappaB-regulated gene expression through direct inhibition of IkappaBalpha kinase beta on cysteine 179 residue. J Biol Chem.

[B55] Pires BRB, Silva R, Ferreira GM, Abdelhay E (2018). NF-kappaB: Two sides of the same coin. Genes (Basel).

[B56] Pires FR, Ramos AB, Oliveira JB (2013). Oral squamous cell carcinoma: clinicopathological features from 346 cases from a single oral pathology service during an 8-year period. J Appl Oral Sci.

[B57] Puar YR, Shanmugam MK, Fan L (2018). Evidence for the Involvement of the Master Transcription Factor NF-kappaB in Cancer Initiation and Progression. Biomedicines.

[B58] Rajendran P, Li F, Manu KA (2011a). gamma-Tocotrienol is a novel inhibitor of constitutive and inducible STAT3 signalling pathway in human hepatocellular carcinoma: potential role as an antiproliferative, pro-apoptotic and chemosensitizing agent. Br J Pharmacol.

[B59] Rajendran P, Li F, Shanmugam MK (2012). Honokiol inhibits signal transducer and activator of transcription-3 signaling, proliferation, and survival of hepatocellular carcinoma cells via the protein tyrosine phosphatase SHP-1. J Cell Physiol.

[B60] Rajendran P, Ong TH, Chen L (2011b). Suppression of signal transducer and activator of transcription 3 activation by butein inhibits growth of human hepatocellular carcinoma in vivo. Clin Cancer Res.

[B61] Ram H, Sarkar J, Kumar H (2011). Oral cancer: risk factors and molecular pathogenesis. J Maxillofac Oral Surg.

[B62] Roy NK, Bordoloi D, Monisha J (2017). Specific targeting of Akt Kinase Isoforms: Taking the precise path for prevention and treatment of cancer. Curr Drug Targets.

[B63] Roy NK, Deka A, Bordoloi D (2016). The potential role of boswellic acids in cancer prevention and treatment. Cancer Lett.

[B64] Sethi G, Chatterjee S, Rajendran P (2014). Inhibition of STAT3 dimerization and acetylation by garcinol suppresses the growth of human hepatocellular carcinoma in vitro and in vivo. Mol Cancer.

[B65] Sethi G, Shanmugam MK, Ramachandran L, Kumar AP, Tergaonkar V (2012). Multifaceted link between cancer and inflammation. Biosci Rep.

[B66] Sethi G, Sung B, Aggarwal BB (2008). Nuclear factor-kappaB activation: from bench to bedside. Exp Biol Med (Maywood).

[B67] Sethi G, Tergaonkar V (2009). Potential pharmacological control of the NF-kappaB pathway. Trends Pharmacol Sci.

[B68] Shanmugam MK, Kannaiyan R, Sethi G (2011a). Targeting cell signaling and apoptotic pathways by dietary agents: role in the prevention and treatment of cancer. Nutr Cancer.

[B69] Shanmugam MK, Lee JH, Chai EZ (2016). Cancer prevention and therapy through the modulation of transcription factors by bioactive natural compounds. Semin Cancer Biol.

[B70] Shanmugam MK, Rajendran P, Li F (2011b). Ursolic acid inhibits multiple cell survival pathways leading to suppression of growth of prostate cancer xenograft in nude mice. J Mol Med (Berl).

[B71] Shanmugam MK, Warrier S, Kumar AP, Sethi G, Arfuso F (2017). Potential role of natural compounds as anti-angiogenic agents in cancer. Curr Vasc Pharmacol.

[B72] Shin EM, Hay HS, Lee MH (2014). DEAD-box helicase DP103 defines metastatic potential of human breast cancers. J Clin Invest.

[B73] Siveen KS, Ahn KS, Ong TH (2014a). Y-tocotrienol inhibits angiogenesis-dependent growth of human hepatocellular carcinoma through abrogation of AKT/mTOR pathway in an orthotopic mouse model. Oncotarget.

[B74] Siveen KS, Mustafa N, Li F (2014b). Thymoquinone overcomes chemoresistance and enhances the anticancer effects of bortezomib through abrogation of NF-kappaB regulated gene products in multiple myeloma xenograft mouse model. Oncotarget.

[B75] Song Z, Shanmugam MK, Yu H, Sethi G (2016). Butein and its role in chronic diseases. Adv Exp Med Biol.

[B76] Subramaniam A, Shanmugam MK, Ong TH (2013). Emodin inhibits growth and induces apoptosis in an orthotopic hepatocellular carcinoma model by blocking activation of STAT3. Br J Pharmacol.

[B77] Tan SM, Li F, Rajendran P (2010). Identification of beta-escin as a novel inhibitor of signal transducer and activator of transcription 3/Janus-activated kinase 2 signaling pathway that suppresses proliferation and induces apoptosis in human hepatocellular carcinoma cells. J Pharmacol Exp Ther.

[B78] Tang CH, Sethi G, Kuo PL (2014). Novel medicines and strategies in cancer treatment and prevention. Biomed Res Int.

[B79] Tang YL, Huang LB, Lin WH (2016). Butein inhibits cell proliferation and induces cell cycle arrest in acute lymphoblastic leukemia via FOXO3a/p27kip1 pathway. Oncotarget.

[B80] Thakur KK, Bordoloi D, Kunnumakkara AB (2018). Alarming burden of triple-negative breast cancer in India. Clin Breast Cancer.

[B81] Thyagarajan A, Sahu RP (2018). Potential contributions of antioxidants to cancer therapy: Immunomodulation and radiosensitization. Integr Cancer Ther.

[B83] Wang E, Wink M (2016). Chlorophyll enhances oxidative stress tolerance in Caenorhabditis elegans and extends its lifespan. Peer J.

[B84] Yang LH, Ho YJ, Lin JF (2012). Butein inhibits the proliferation of breast cancer cells through generation of reactive oxygen species and modulation of ERK and p38 activities. Mol Med Rep.

[B85] Yang PY, Hu DN, Kao YH, Lin IC, Liu FS (2018). Butein induces apoptotic cell death of human cervical cancer cells. Oncol Lett.

[B86] Yang SF, Weng CJ, Sethi G, Hu DN (2013). Natural bioactives and phytochemicals serve in cancer treatment and prevention. Evid Based Complement Alternat Med.

[B87] Yarla NS, Bishayee A, Sethi G (2016). Targeting arachidonic acid pathway by natural products for cancer prevention and therapy. Semin Cancer Biol.

[B88] Zarghi A, Arfaei S (2011). Selective COX-2 Inhibitors: A review of their structure-activity relationships. Iran J Pharm Res.

[B89] Zhang L, Chen W, Li X (2008). A novel anticancer effect of butein: inhibition of invasion through the ERK1/2 and NF-kappa B signaling pathways in bladder cancer cells. FEBS Lett.

[B90] Zhou Y, Li M, Yu X (2018). Butein suppresses hepatocellular carcinoma growth via modulating Aurora B kinase activity. Int J Biol Sci.

